# Molecular taxonomy confirms that the northeastern Atlantic and Mediterranean Sea harbor a single lancelet, *Branchiostoma lanceolatum* (Pallas, 1774) (Cephalochordata: Leptocardii: Branchiostomatidae)

**DOI:** 10.1371/journal.pone.0251358

**Published:** 2021-05-06

**Authors:** Filomena Caccavale, David Osca, Salvatore D’Aniello, Fabio Crocetta

**Affiliations:** 1 Department of Biology and Evolution of Marine Organisms, Stazione Zoologica Anton Dohrn Napoli, Naples, Italy; 2 Department of Integrated Marine Ecology, Stazione Zoologica Anton Dohrn Napoli, Naples, Italy; Laboratoire de Biologie du Développement de Villefranche-sur-Mer, FRANCE

## Abstract

Branchiostomatidae (lancelets or amphioxus) comprises about 30 species, several of which are well-established models in evolutionary development. Our zoological and ecological knowledge of the family is nonetheless limited. Despite evident differences can be found among known populations, the taxonomy of *Branchiostoma lanceolatum* (type species of the genus *Branchiostoma*) has never been investigated with modern methods through its range in the northeastern Atlantic and Mediterranean Sea. We address this via a multilocus molecular approach and comparing specimens collected from different European populations. Results obtained here confirm the presence of a single species inhabiting the range between the topotypical localities of *B*. *lanceolatum* (Atlantic Ocean) and of its junior synonym *B*. *lubricum* (Mediterranean Sea), without evincing geographical structure between populations. This suggests that environment most likely drives the characteristics observed in different geographic areas. The long larval phase and the slow mutation rate in lancelets may have played a key role in the evolutionary history of this iconic species.

## Introduction

The family Branchiostomatidae Bonaparte, 1846 (subphylum Cephalochordata) comprises about thirty species, known as lancelets or amphioxus [1–4]. They inhabit the soft bottoms of various sublittoral and coastal habitats (estuaries, coastal lagoons, river deltas, and open coasts) from temperate to tropical regions [5–7] and some species grow up to 10 cm in length. Lancelets are generally benthic, living half-buried and only exposing the rostral end to the water. They filter plankton through the gill-bars by generating a ciliary water current, entering from the mouth through the buccal cirri. Food particles are embedded in mucus, collected in the pharynx and then passed into the intestine [6–10].

Until recently, lancelets were divided in the genera *Branchiostoma* Costa, 1834 (the most diverse genus, exceeding twenty species) and *Epigonichthys* Peters, 1876 [1, 11]. However, subsequent studies reinstated the genus *Asymmetron* Andrews, 1893; these allocated there some taxa previously ascribed to *Epigonichthys* and investigated the phylogenetic relationships between the three clades, suggesting that *Asymmetron* diverged first and that *Epigonichthys* and *Branchiostoma* are sister groups [2, 3, 12–14].

Notwithstanding morphological differences between the three genera in gonads organization, metapleural fold, and caudal process [1, 2, 14], they share the same adult morphology, a translucent and elongated body with well visible neural tube, a notochord, an endostyle, a segmented musculature and a postanal tail [15–17]. Despite sharing these features with vertebrates, lancelets lack key vertebrate structures, such as migratory neural crest cells, a highly regionalized brain, or paired sense organs [18].

The morphological similarity of lancelets to vertebrates and their phylogenetic relatedness has attracted the scientific attention of biologists for centuries [19]. In particular, the European *Branchiostoma lanceolatum* [20], the East Asian *Branchiostoma belcheri* [21], and the Floridian–Caribbean *Branchiostoma floridae* Hubbs, 1922, have become established model organisms for the evolution of the developmental mechanisms (Evo-Devo) during the transition from invertebrate to vertebrate chordates [16, 22–25]. Moreover, the lancelet genome resembles that of the chordate ancestor in terms of conserved organization, regulation, and function [26–28].

Despite the general importance of Branchiostomatidae, little is known about much of this family. Several species were newly described, or their taxonomy has been clarified only recently [2, 29, 30], and species misidentification or cryptic diversity have been found using molecular approaches or integrative taxonomy [14, 31–36]. Moreover, new records of lancelet larvae or adults improved our assessment of species-specific geographical distribution and ecological traits at a range of scales [37–46].

Finally, even widely studied lancelet species still lack rigorous characterization. As an example, the taxonomy and phylogeography of *B*. *lanceolatum*, the type species of its genus (see [47]), has never been investigated with modern approaches through its range in the northeastern Atlantic–Mediterranean Sea. Yet, populations differ in size and morphology (Atlantic specimens are larger), developmental rate (Atlantic larvae grow slower), and spawning period (of longer duration in the Mediterranean Sea) [48–51]. We addressed this using a multilocus molecular approach to compare *B*. *lanceolatum* specimens collected from diverse European populations by both Atlantic and Mediterranean coasts.

## Material and methods

### Sampling

**Fig 1:**
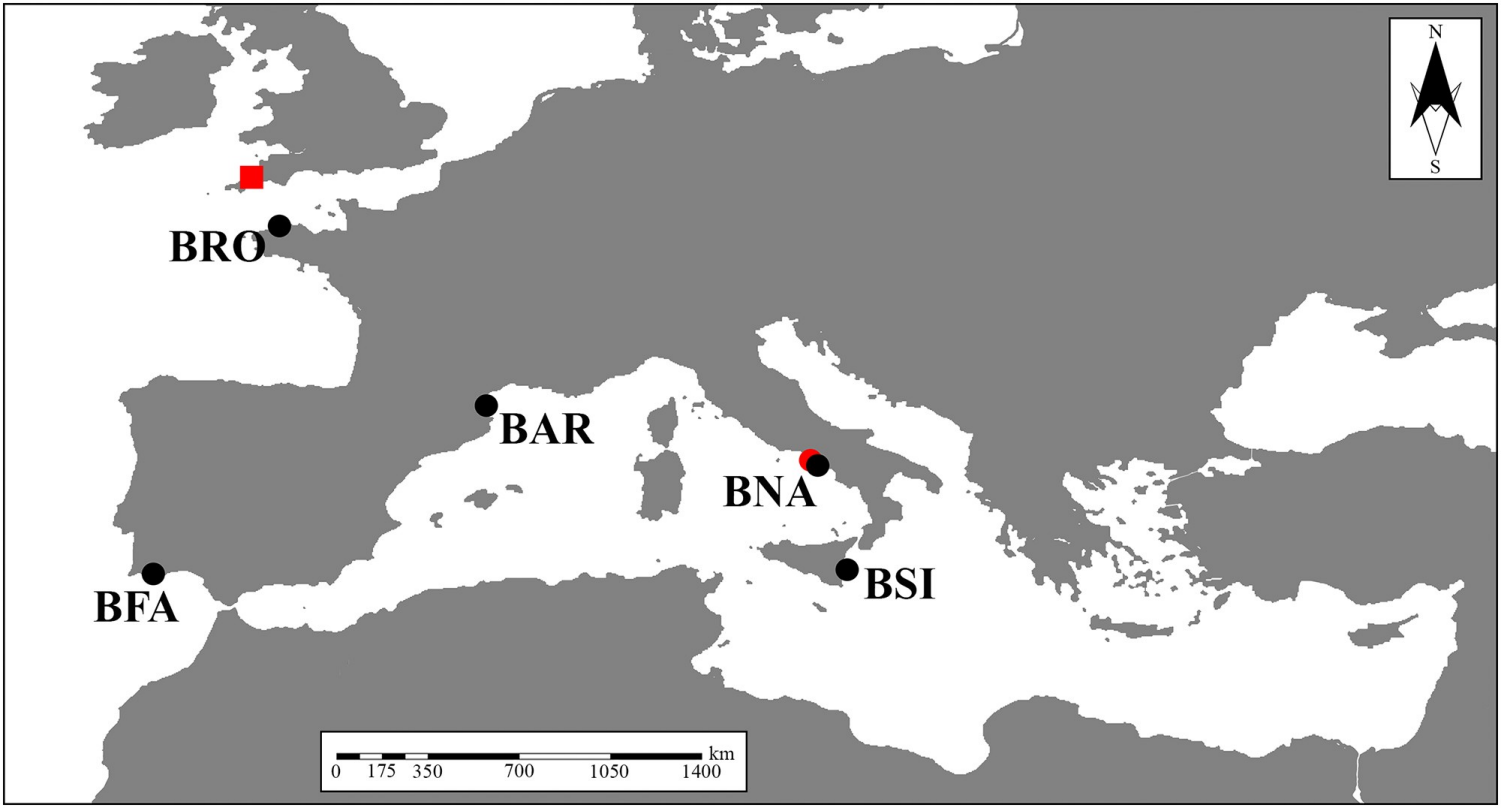
Map of the sampling sites (codes correspond to the localities reported in Table 1) highlighting the type localities of *Limax lanceolatus* Pallas, 1774 (red square) and *Branchiostoma lubricum* Costa, 1834 (red circle).

Lancelet specimens were collected between 2012 and 2017 in five European localities (two from the Atlantic Ocean, two from the western Mediterranean Sea, and one from the central-eastern Mediterranean Sea) and including populations widely exploited for Evo-Devo studies [28, 52–56]. Noteworthy, specimens were also sampled from near the type localities of *Limax lanceolatus* Pallas, 1774 (Cornwall: see [20]) and *Branchiostoma lubricum* Costa, 1834 (Naples, Italy: see [47]), the only confirmed subjective junior synonym of *B*. *lanceolatum*. Sampled localities are summarized in Table 1 and shown in Fig 1. Voucher specimens were fixed in 70–100% ethanol for molecular analyses, shortly after collection.

**Table 1:** Sampling sites (codes as in Fig 1) with geographic coordinates (WGS 84), environmental data, sampling gear, date, and *legit*.

N	Locality	Coordinates	Substrate	Sampling	Date	*Legit*
BRO	France: Roscoff	48.726667, -3.850833	gravel, 1–2 m	dredge	May 2017	Agnés Boutet
BFA	Portugal: Faro, Ria Formosa	37.009093, -7.995101	sand, 1–2 m	hand dredge	July 2012	Filipe Castro
BAR	France: Argelès sur Mer, Le Racou	42.540802, 3.061389	sand, 8–15 m	dredge	June 2016	Hector Escriva
BNA	Italy: Napoli, Posillipo	40.809354, 14.208846	sand, 8–15 m	dredge	May 2015	Salvatore D’Aniello
BSI	Italy: Siracusa, Plemmirio MPA	37.039364, 15.309600	sand, 10–12 m	grab	June 2015	Gianfranco Mazza

### Ethical statement

The field study did not involve endangered or protected species. All animal procedures were in compliance with the European Union guidelines.

### DNA extraction, amplification, and sequencing

Genomic DNA was extracted from adult lancelets as previously described [56]. Partial sequences of three mitochondrial genes widely used for taxonomic studies, namely *cytochrome c oxidase subunit I* (COX1), *12S ribosomal ribonucleic acid* (12S rRNA), and *16S ribosomal ribonucleic acid* (16S rRNA), were amplified from three specimens randomly selected from each sampling locality, using the following species-specific primers designed for this study using OligoEvaluator^™^: Cox1_forward 5’-GATTCATAATATGCGTGCTAGC-3’ and Cox1_reverse 5’-CGGCTCCTATAGACAAAACG-3’; 12S_forward 5’-GGGTTACTGATGATACATGC-3’ and 12S_reverse 5’-CTACTATTGACTACACCCTG-3’; 16S_forward 5’-CGCCTGTTTAACAAAAACAT-3’ and 16S_reverse 5’-CGGTCTGTACTCAGATCACGTA-3’.

PCR was conducted in a 25 μl volume reaction, containing 5 μl of Green GoTaq Reaction Buffer (5×), 1.75 μl of MgCl_2_ (25 mM), 0.5 μl dNTP mix (10 mM each), 1 μl of template DNA (50–80 ng/μl), 2.5 μl of each primer (5 μM), 0.12 μl of GoTaq^®^ DNA Polymerase (5 u/μl), and distilled water. Amplifications were performed according to the following conditions: initial denaturation at 95°C (5 min), followed by 30 cycles of denaturation at 95°C (30 sec), annealing at 55°C (30 sec), extension at 72°C (1 min), with a final extension at 72°C (5 min).

PCR products were examined on ethidium bromide-stained 1% agarose-TAE gels, and bands of the appropriate molecular weight were extracted from the gel and purified using the GFX^™^ PCR DNA and Gel Band Purification Kit (GE Healthcare Life Sciences). For each specimen, the three amplified gene fragments were cloned in pGEM^®^-T Easy Vector (Promega) and Sanger sequenced from both directions using the M13 forward and reverse primers (Promega). Sequencing was carried out with an Automated Capillary Electrophoresis Sequencer 3730 DNA Analyzer (Applied Biosystems) using the BigDye^®^ Terminator v3.1 Cycle Sequencing Kit (Life Technologies).

### Sequences and sequence alignment

Sequences obtained for the three gene fragments were compared with reference sequences from the NCBI nucleotide database using BLASTn [57], and assembled into single contigs for each specimen using Sequencher v.5.4 (Gene Codes Co.; Ann Arbor, MI, USA). Complete mitochondrial genomes from the genus *Branchiostoma* were acquired from GenBank [58], together with those of *Epigonichthys maldivensis* (NC_006465) as outgroup based on its sister relationship with *Branchiostoma* taxa [3, 14, 36].

To construct the data set, the amino acid sequences of the partial COX1 gene were aligned using Translator X [59] to better align based on peptide sequence, whereas orthologous nucleotide sequences of the ribosomal RNA mitochondrial genes were aligned separately using MAFFT [60]. The nucleotide alignments from the three gene fragments (COX1, 12S, and 16S) were then concatenated. Alignment format conversions were performed using the ALTER webserver [61].

### Phylogenetic analyses

We conducted phylogenetic analyses on the complete aligned and concatenated multilocus data sets, using two optimality criteria: Maximum likelihood estimation (ML) [62] and Bayesian inference (BI) [63]. ML analyses were conducted with RAxML v.8.1.16 [64] using the rapid hill-climbing algorithm.

BI analyses were conducted using MrBayes v.3.2.7a [65] on XSEDE through the on-line CIPRES Science Gateway v.3.3 [66]. We ran four simultaneous Markov chains for two million generations, sampling every thousand generations, and discarding the first quarter of generations, to prevent sampling before reaching stationarity (assessed by plots of log likelihood values and standard deviation of split frequencies). Two independent Bayesian inference runs were performed to assure adequate mixing of the Markov chains and detect convergence.

The best partition scheme and best-fit models of substitution for the data set were identified with Partition Finder 2 [67], applying the Akaike information criterion [68]. The partitions tested were all genes combined; all genes separated; and genes grouped by subunits—i.e. cox, ribosomal. Support for internal branches was evaluated by non-parametric bootstrapping [69] with a thousand replicates (ML) and by Bayesian posterior probabilities (BI). Maximal, high, moderate, and poor support for nodes was defined for ML as 100%, >70%, 50–70%, and below 50%, and for BI as 1, >0.95, 0.90–0.95%, 0.90, respectively.

### Genetic distances

We evaluated genetic distances to assess species boundaries. Genetic distances (shown as the percentage difference in base substitutions per sites) were computed using the Kimura 2-Parameters (K2P) model [70] with a thousand bootstrap resampling using MEGA X [71], treating the concatenated dataset (COX1, 12S, and 16S) as a single locus.

### Species delimitation analyses

Three different approaches were used for species delimitation: the Bayesian implementation of the Poisson Tree Processes model (bPTP) [72], the Automatic Barcode Gap Discovery (ABGD) method [73], and a statistical parsimony network analysis (TCS) [74].

The bPTP is a phylogeny-based species delimitation method that delimits species based on single-locus molecular data [72]. Species delimitation was thus analyzed using the entire mitochondrial dataset (COX1+12S+16S) as a single-locus data [75], and the bPTP was run to compare the number of species delimited by each model. The PTP model uses non-ultrametric trees to enumerate species in terms of the number of substitutions, which indicates branch length. The phylogenetic trees obtained using BI and ML provided inputs for comparison.

The bPTP analysis was performed in the Exelixis Lab species delimitation web server (http://species.h-its.org), as follows: the number of MCMC generations was 10^5^, as recommended for small trees, thinning was set to 100 and we discarded the first quarter of samples. Convergence of the parameters was checked after the run.

The ABGD method detects the so-called ‘barcode gap’ in the distribution of pairwise distances [73, 76]. A distance value corresponding to the most reliable gap was used to group the sequences in MOTUs (Molecular Operational Taxonomic Units) [77]. The concatenated dataset alignment was processed using the ABGD program (https://bioinfo.mnhn.fr/abi/public/abgd/abgdweb.html), excluding the outgroup *E*. *maldivensis*. We applied default parameters with the Kimura two-parameter (K2P) model [70] and the following settings: a prior constraint on the maximum value of intraspecific divergence between 0.001 and 0.1, 10 recursive steps within the primary partitions defined by the first estimated gap, and a gap width of 1.5.

The statistical parsimony network analysis calculates the maximum number of mutational steps constituting a parsimonious connection between two haplotypes [74, 78]. The haplotypes are then joined into networks as per Templeton and colleagues [79], and those separated by more mutational steps (i.e. probability of secondary mutations exceeding 5%) remain disconnected. A statistical parsimony network analysis implemented in the TCS program [80] was applied to the complete *Branchiostoma* dataset to differentiate species in a mixed sample [81, 82]. Moreover, two complementary analyses (one with the concatenated fragments and one with the COX1 fragment only) were also carried out on the *B*. *lanceolatum* dataset to identify any geographic structure within the haplotypes found.

## Results

Partial sequences of the three mitochondrial genes [COX1 (621 bp), 12S (488 bp), and 16S (501 bp)] were obtained from three specimens from each of the five different localities (Fig 1, Table 1, S1 Table). These were deposited in GenBank and accession numbers are reported in S2 Table. Our GenBank data extraction yielded complete mitochondrial genomes of 48 *Branchiostoma* specimens, belonging to four different taxa, as well as that of *E*. *maldivensis* (S1 Table).

**Fig 2:**
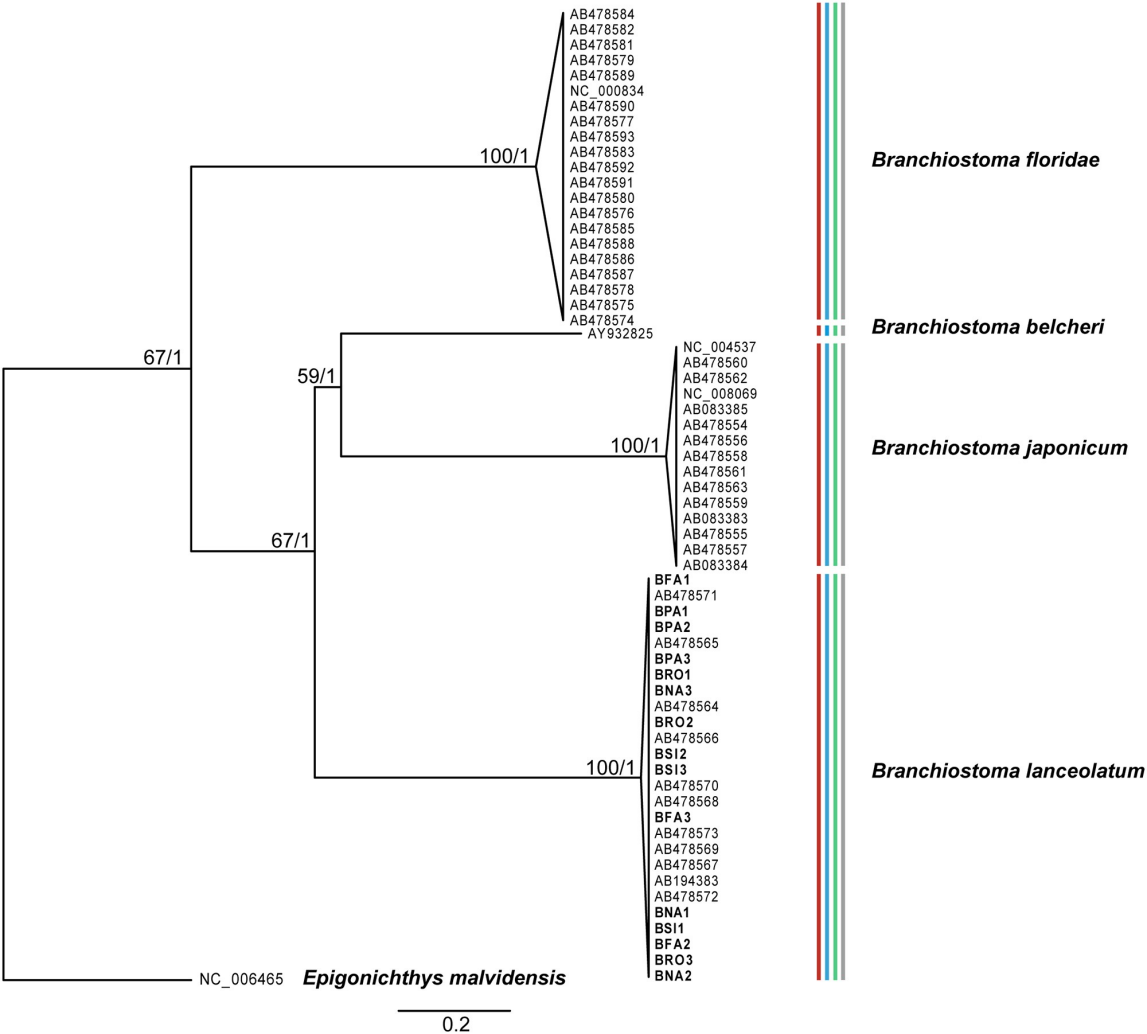
ML and BI consensus tree based on the analyses of the concatenated mitochondrial dataset (COX1+12S+16S). Values at nodes represent ML bootstrap support and Bayesian posterior probabilities, respectively. Colored bars indicate the results of the species delimitation analyses: bPTP with ML tree in red, bPTP with Bayesian tree in blue, ABGD in green, and TCS in grey. Novel sequence samples are highlighted in bold (see S1 Table for codes).

We acquired fragments of all the three genes from all complete mitochondrial genomes derived from GenBank, and generated a concatenated matrix including all specimens. After alignment, the sequence data used for analyses consisted of 1653 characters (S1 File). The best partition scheme for the data set was to treat the three concatenated loci separately. The best-fit model of substitution for all was the GTR+G model. ML (-ln L = 7718.59) and BI (-ln L = 8331.61 and -ln L = 8341.92 for the respective runs) analyses reached a similar tree topology, with four well-defined terminal clades gaining maximal support (Fig 2).

Relationships between *Branchiostoma* clades were unclear, showing maximal support for the BI analysis, but moderate support for the ML analysis (Fig 2). Moreover, our results placed *B*. *floridae* as the sister species of a group composed by the remaining species, with *B*. *lanceolatum* diverging first, and *B*. *belcheri* and *B*. *japonicum* as sister species. Such data are consistent with a previous work by [83] who used protein-coding genes of the mitochondrial DNA, although they differ from other studies using phylogenomic data [84], complete mitochondrial genomes [33], and 12S rRNA [83]. Phylogenetic relationships based on complete mitochondrial genomes are presumably more reliable than those obtained here; however, this is out of the scope of the present work.

At a species level, two of the four terminal clades were monophyletic (those representing *B*. *floridae* and *B*. *lanceolatum*) and the clade representing *B*. *lanceolatum* included all our experimentally-derived specimens studied here. The two remaining clades comprised (i) a single specimen of *B*. *belcheri* (AY932825: [83]) and (ii) several specimens described as *B*. *belcheri* clustering with a single specimen of *B*. *japonicum* (NC_008069, derived from DQ407722: [83]), respectively (Fig 2; S1 Fig). This first clade (i) corresponds to *B*. *belcheri*, whereas the latter (ii) corresponds to *B*. *japonicum*, which was considered a junior synonym or a subspecies of *B*. *belcheri* until recently [13, 83].

The species delimitation approaches arrived at similar results and confirmed the topology of the phylogenetic analyses, yielding four well-defined MOTUs (Fig 2; S1 Fig). The TCS analysis defined a total of 61 haplotypes in the concatenated dataset. Two *B*. *lanceolatum* (AB194383 and AB478572) and two *B*. *floridae* (AB478581 and AB478582) specimens, respectively, had identical sequences in all three fragments.

All the interspecific genetic distances were over 20% (Table 2). The lower genetic distance was found between *B*. *lanceolatum* and *B*. *belcheri* (mean 20.6%, SEM ± 1.2%), while the highest was between *B*. *floridae* and *B*. *japonicum* (mean 25.9%, SEM ± 1.4%).

**Table 2:** Average of the uncorrected pairwise genetic distances between *Branchiostoma* species based on the concatenated dataset (COX1, 12S, and 16S).

	***B*. *belcheri***	***B*. *japonicum***	***B*. *floridae***
***B*. *japonicum***	21.2 ± 1.2		
***B*. *floridae***	24.1 ± 1.3	25.9 ± 1.4	
***B*. *lanceolatum***	20.6 ± 1.2	22.7 ± 1.3	23.5 ± 1.4

Values reported as percentage ± mean standard error.

**Fig 3:**
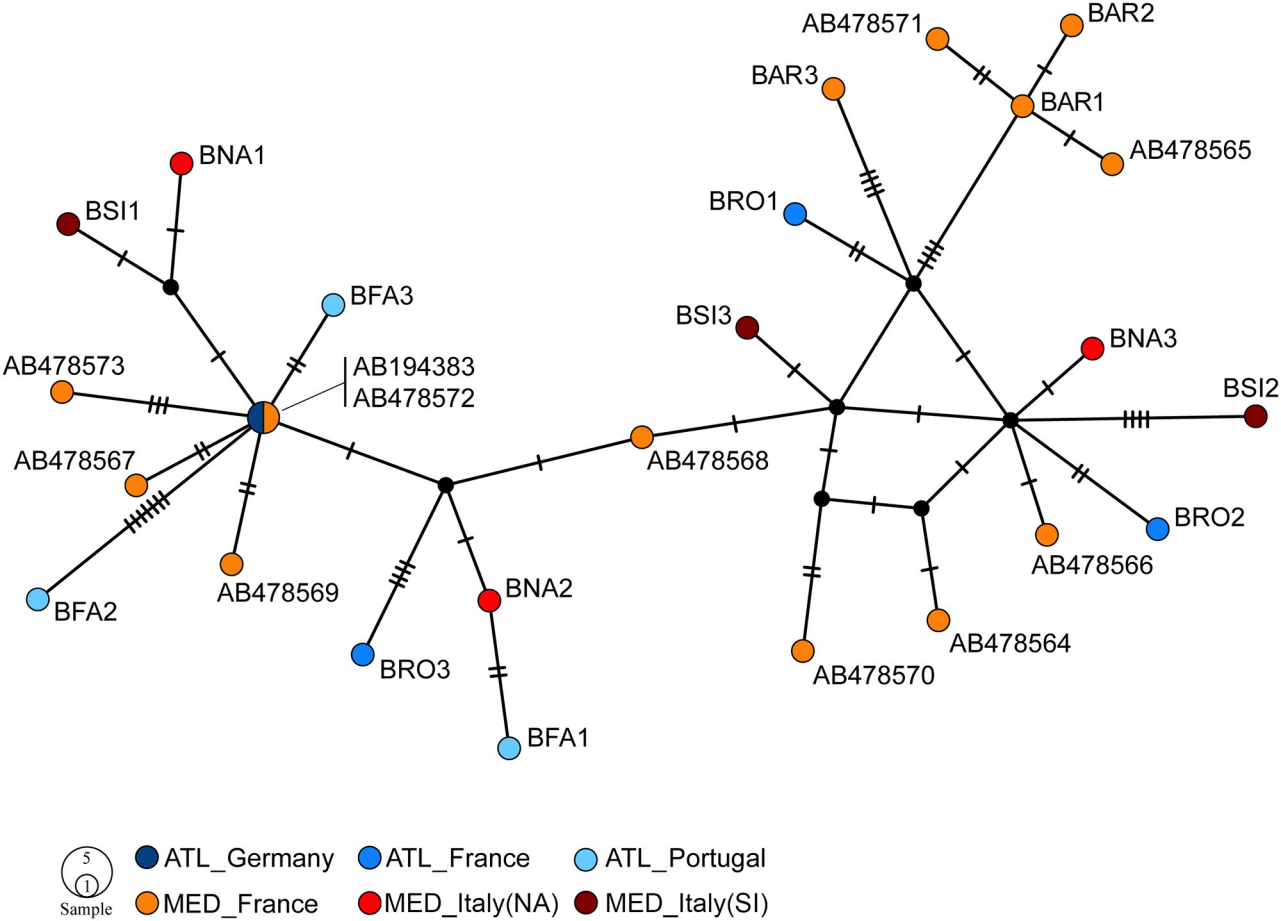
TCS for the concatenated matrix (COX1+12S+16S) of *B*. *lanceolatum*, showing relationships between the recorded haplotypes. See S1 Table for codes. Abbreviations used: ATL–Atlantic Ocean; MED–Mediterranean Sea; AR–Argelès sur Mer; NA–Napoli; SI–Siracusa. Circles representing haplotypes are scaled to their frequencies. Black dots represent missing intermediate haplotypes. Branch length connecting the different haplotypes is proportional to the number of mutations, with small transversal lines along the connecting branches representing mutational steps.

The TCS analysis on the concatenated *B*. *lanceolatum* dataset yielded a total of 25 different haplotypes found in the 26 specimens analyzed (AB194383 and AB478572 were identical, see above), but found no clear geographic structure (Fig 3). A similar result was also obtained when analyzing the COX1 fragment only, with 17 haplotypes and the following samples sharing haplotypes: (i) AB194383, AB478567, AB478572, AB478573, and BFA3; (ii) BNA2, BFA1, and BRO3; (iii) AB478568, AB478570, and BSI3; (iv) AB478571 and BAR1 (S2 Fig). In both cases, the Mediterranean coast of France had the most haplotypes (13 in the concatenated dataset and 9 in the COX1 matrix), possibly explained by this region contributing the most samples.

## Discussion

The Mediterranean marine biogeography is mostly a subset of that of the Atlantic, having originated with the re-establishment of the Atlantic–Mediterranean connection (5.33 million years ago) [85, 86], a phenomenon that would suggest conspecificity between the biotas of the two seas. Indeed, several species possess an Atlantic–Mediterranean distribution [87–90]. However, phylogeographical barriers between the Atlantic Ocean and the Mediterranean Sea and the geographical complexity of the Mediterranean have given rise to endemism, and cryptic and vicariant species, even among conspicuous species and model systems [82, 91–94].

Speciation remains one of the most controversial and least understood topics in evolution due to the complexity and diversity of living organisms; it typically results from combining various intrinsic and extrinsic factors including geographic barriers to larval dispersal, duration of the larval phase, and reproductive isolation due to prezygotic and postzygotic mechanisms [95–98].

Recent advances in the use of DNA barcoding and integrative taxonomy are now clarifying relationships between species and within ambiguous species-groups, including the biota of the northeastern Atlantic and the Mediterranean Sea [92–94, 99, 100]. In fact, while the systematics of the Atlantic–Mediterranean biota dates back centuries [85, 86, 101, 102], we need to test historical and morphological taxonomy with modern research approaches. This is particularly important when differences among species can be subtle, as exemplified by Branchiostomatidae. Lancelets are typical examples of morphological and evolutionary stasis, exhibiting conserved morphology, genetic machinery, and development regulation, even in species that diverged million of years ago [103, 104].

Despite we tested here *B*. *lanceolatum* specimens from throughout its range in the northeastern Atlantic and the Mediterranean, none of the analyses done was able to discern them at the species level, which implies that they constitute a single species. This suggests that environment most likely drives the peculiar characteristics observed in certain populations. Indeed in diverse taxonomic groups, the Atlantic specimens outgrow their Mediterranean counterparts [105], whereas developmental rates and spawning periods may differ due to the discrepant mean temperatures between the two seas. Finally, the TCS analysis also failed to identify a clear geographic structure; while this method warrants further testing with more samples, it is indeed in agreement with studies of other lancelet populations worldwide [106, 107].

The absence of speciation at the Atlantic–Mediterranean boundary may be explained by (among other factors) the long-duration of the larval phase before settlement and the sluggish mutation rate of this clade. In fact, the planktonic larvae of *B*. *lanceolatum* dwell in the plankton until metamorphosis [48, 51], and the duration of the larval phase of lancelets is long, varying from one to three months, depending on species [9, 108–110]. This species may therefore disperse widely, enhancing genetic connectivity among distant populations, and thus diminishing population structure. Moreover, cephalochordates generally exhibit a slow mutation rate, with diverse *Branchiostoma* species barely differing even within their complete mitochondrial sequences [111–113].

Notwithstanding present results, this work refines our taxonomic and phylogeographic understanding of an iconic and important model species and, more generally, of the Atlantic–Mediterranean biota. Given that *B*. *lanceolatum* is the most widely-distributed species in the genus, with historical records from other biogeographic areas than the Atlantic–Mediterranean, including the Red Sea and the Indo–Pacific region (review in [1]), further work ought to explore the taxonomy of this species in A Phylogenomic Framework and Divergence History of Cephalochordata Amphioxus Framework global context.

## Supporting information

S1 Table GenBank identification numbers for Branchiostoma and *Epigonichthys maldivensis* sequences used in the present analyses and associated specimen data (localities obtained from GenBank and/or relevant paper/s). *Misidentifications for *Branchiostoma japonicum* (see [13, 83]). Codes as in Table 1, Figs 1–3 and S1 and S2 Figs. Abbreviations used (GenBank ID): CM—complete mitochondrial; COX1—*cytochrome c oxidase subunit I*; 12S - *12S ribosomal ribonucleic acid*; 16S - *16S ribosomal ribonucleic acid*.
(PDF)

S2 Table GenBank accession numbers. (XLSX)

S1 Fig TCS for the concatenated matrix (COX1+12S+16S) of *Branchiostoma lanceolatum*, showing relationships between the recorded haplotypes. See S1 Table for codes. Circles representing haplotypes are scaled to their frequencies. Branch length connecting the different haplotypes is proportional to the number of mutations, with small transversal lines along the connecting branches representing mutational steps.
(TIF)

S2 Fig TCS for the COX1 matrix of *Branchiostoma lanceolatum*, showing relationships between the recorded haplotypes. See S1 Table for codes. Abbreviations used: ATL–Atlantic Ocean; MED–Mediterranean Sea; NA–Napoli; SI–Siracusa. Circles representing haplotypes are scaled to their frequencies. Black dots represent missing intermediate haplotypes. Branch length connecting the different haplotypes is proportional to the number of mutations, with small transversal lines along the connecting branches representing mutational steps.
(TIF)

S1 File Alignment of the concatenated dataset (COX1+12S+16S). (FAS)
