# Supplementary material for: Molecular taxonomy confirms that the northeastern Atlantic and Mediterranean Sea harbor a single lancelet, Branchiostoma lanceolatum (Pallas, 1774) (Cephalochordata: Leptocardii: Branchiostomatidae)
Source: PLoS One. 2021 May 6;16(5):e0251358. doi: 10.1371/journal.pone.0251358 (PMC8101936; doi:10.1371/journal.pone.0251358)
Supplement: S1 Table — *Misidentifications for Branchiostoma japonicum (see [13, 83]). Codes as in Table 1, Figs 1–3 and S1 and S2 Figs. Abbreviations used (GenBank ID): CM—complete mitochondrial; COX1—cytochrome c oxidase subunit I; 12S - 12S ribosomal ribonucleic acid; 16S - 16S ribosomal ribonucleic acid. (PDF) [file pone.0251358.s001.pdf]

S1 Table

Species	Locality	CODES	GenBank ID				Reference
			CM	COX1	12S	16S	
B. belcheri	China: Xiamen		AY932825				Zhong et al. (2009)
*B. belcheri**	Japan: Sea of Genkai		AB083383				Takada et al. (2004)
*B. belcheri**	Japan: Akashi		AB083384				Takada et al. (2004)
*B. belcheri**	Japan: Akashi		AB083385				Takada et al. (2004)
*B. belcheri**	Japan: Sea of Genkai		AB478554				GenBank
*B. belcheri**	Japan: Sea of Genkai		AB478555				GenBank
*B. belcheri**	Japan: Sea of Genkai		AB478556				GenBank
*B. belcheri**	Japan: Sea of Genkai		AB478557				GenBank
*B. belcheri**	Japan: Sea of Genkai		AB478558				GenBank
*B. belcheri**	Japan: Sea of Genkai		AB478559				GenBank
*B. belcheri**	Japan: Sea of Genkai		AB478560				GenBank
*B. belcheri**	Japan: Sea of Genkai		AB478561				GenBank
*B. belcheri**	Japan: Sea of Genkai		AB478562				GenBank
*B. belcheri**	Japan: Sea of Genkai		AB478563				GenBank
*B. belcheri**	Japan: Sea of Genkai		NC_004537				Takada et al. (2004)
B. floridae	Florida: Tampa Bay		AB478574				GenBank
B. floridae	Florida: Tampa Bay		AB478575				GenBank
B. floridae	Florida: Tampa Bay		AB478576				GenBank
B. floridae	Florida: Tampa Bay		AB478577				GenBank
B. floridae	Florida: Tampa Bay		AB478578				GenBank
B. floridae	Florida: Tampa Bay		AB478579				GenBank
B. floridae	Florida: Tampa Bay		AB478580				GenBank
B. floridae	Florida: Tampa Bay		AB478581				GenBank
B. floridae	Florida: Tampa Bay		AB478582				GenBank
B. floridae	Florida: Tampa Bay		AB478583				GenBank
B. floridae	Florida: Tampa Bay		AB478584				GenBank
B. floridae	Florida: Tampa Bay		AB478585				GenBank
B. floridae	Florida: Tampa Bay		AB478586				GenBank
B. floridae	Florida: Tampa Bay		AB478587				GenBank
B. floridae	Florida: Tampa Bay		AB478588				GenBank

B. floridae	Florida: Tampa Bay		AB478589				GenBank
B. floridae	Florida: Tampa Bay		AB478590				GenBank
B. floridae	Florida: Tampa Bay		AB478591				GenBank
B. floridae	Florida: Tampa Bay		AB478592				GenBank
B. floridae	Florida: Tampa Bay		AB478593				GenBank
B. floridae	Florida (presumably)		NC_000834				Boore et al. (1999)
B. japonicum	China: Xiamen		NC_008069				Zhong et al. (2009)
B. lanceolatum	Germany: Helgoland		AB194383				Nohara et al. (2005)
B. lanceolatum	France: Mediterranean coast		AB478564				GenBank
B. lanceolatum	France: Mediterranean coast		AB478565				GenBank
B. lanceolatum	France: Mediterranean coast		AB478566				GenBank
B. lanceolatum	France: Mediterranean coast		AB478567				GenBank
B. lanceolatum	France: Mediterranean coast		AB478568				GenBank
B. lanceolatum	France: Mediterranean coast		AB478569				GenBank
B. lanceolatum	France: Mediterranean coast		AB478570				GenBank
B. lanceolatum	France: Mediterranean coast		AB478571				GenBank
B. lanceolatum	France: Mediterranean coast		AB478572				GenBank
B. lanceolatum	France: Mediterranean coast		AB478573				GenBank
B. lanceolatum	France: Argèles-sur-Mer	BAR1		XXXXXXX	XXXXXXX	XXXXXXX	This study
B. lanceolatum	France: Argèles-sur-Mer	BAR2		XXXXXXX	XXXXXXX	XXXXXXX	This study
B. lanceolatum	France: Argèles-sur-Mer	BAR3		XXXXXXX	XXXXXXX	XXXXXXX	This study
B. lanceolatum	France: Roscoff	BRO1		XXXXXXX	XXXXXXX	XXXXXXX	This study
B. lanceolatum	France: Roscoff	BRO2		XXXXXXX	XXXXXXX	XXXXXXX	This study
B. lanceolatum	France: Roscoff	BRO3		XXXXXXX	XXXXXXX	XXXXXXX	This study
B. lanceolatum	Portugal: Faro	BFA1		XXXXXXX	XXXXXXX	XXXXXXX	This study
B. lanceolatum	Portugal: Faro	BFA2		XXXXXXX	XXXXXXX	XXXXXXX	This study
B. lanceolatum	Portugal: Faro	BFA3		XXXXXXX	XXXXXXX	XXXXXXX	This study
B. lanceolatum	Italy: Napoli	BNA1		XXXXXXX	XXXXXXX	XXXXXXX	This study
B. lanceolatum	Italy: Napoli	BNA2		XXXXXXX	XXXXXXX	XXXXXXX	This study
B. lanceolatum	Italy: Napoli	BNA3		XXXXXXX	XXXXXXX	XXXXXXX	This study
B. lanceolatum	Italy: Siracusa	BSI1		XXXXXXX	XXXXXXX	XXXXXXX	This study
B. lanceolatum	Italy: Siracusa	BSI2		XXXXXXX	XXXXXXX	XXXXXXX	This study
B. lanceolatum	Italy: Siracusa	BSI3		XXXXXXX	XXXXXXX	XXXXXXX	This study
E. maldivensis	Japan: Okinawa		NC_006465				Nohara et al. (2005)
