# Supplementary figures and images for: Molecular taxonomy confirms that the northeastern Atlantic and Mediterranean Sea harbor a single lancelet, Branchiostoma lanceolatum (Pallas, 1774) (Cephalochordata: Leptocardii: Branchiostomatidae)
Source: PLoS One. 2021 May 6;16(5):e0251358. doi: 10.1371/journal.pone.0251358 (PMC8101936; doi:10.1371/journal.pone.0251358)

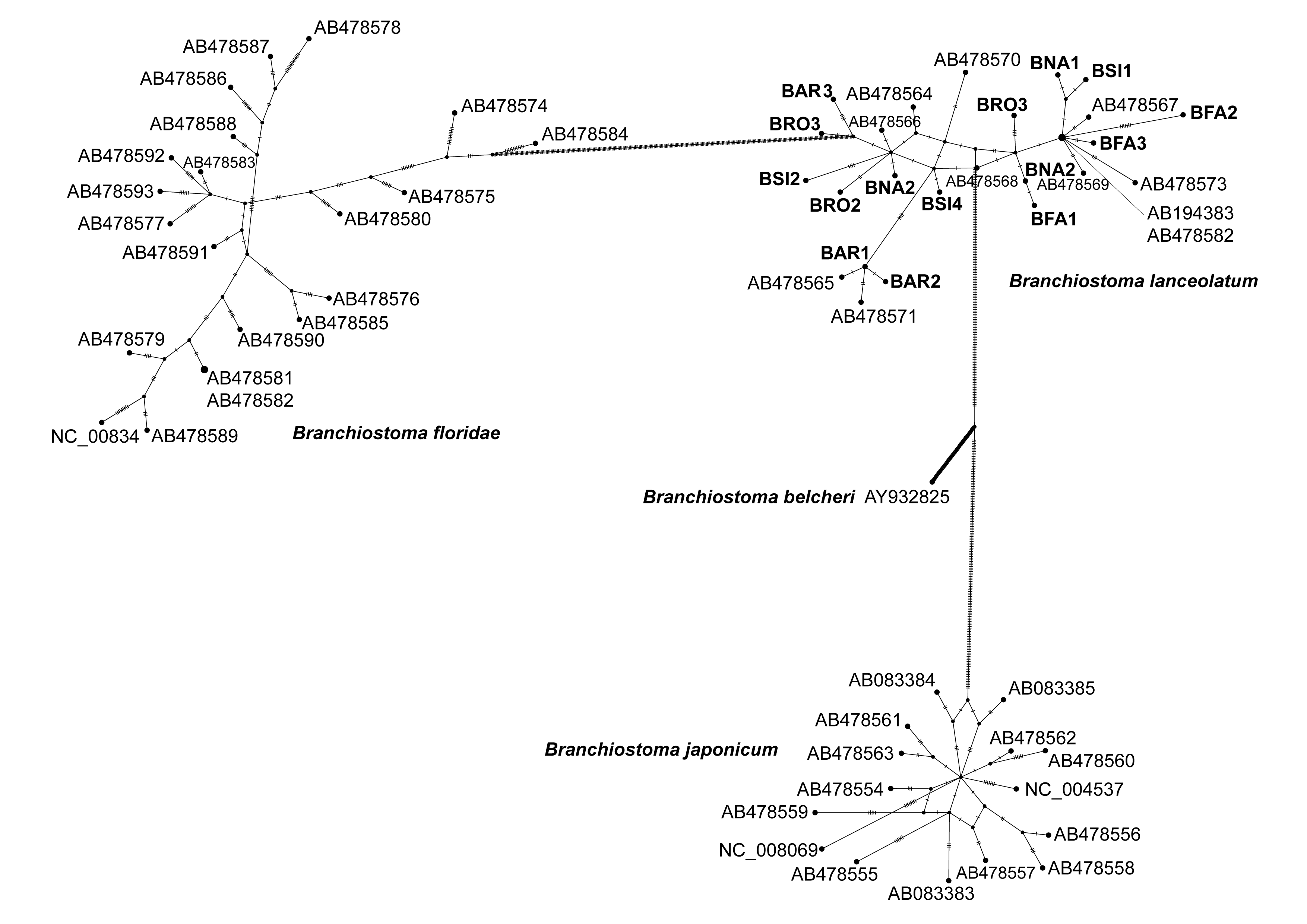

Supplement: S1 Fig — See S1 Table for codes. Circles representing haplotypes are scaled to their frequencies. Branch length connecting the different haplotypes is proportional to the number of mutations, with small transversal lines along the connecting branches representing mutational steps. (TIF) [file pone.0251358.s003.tif]

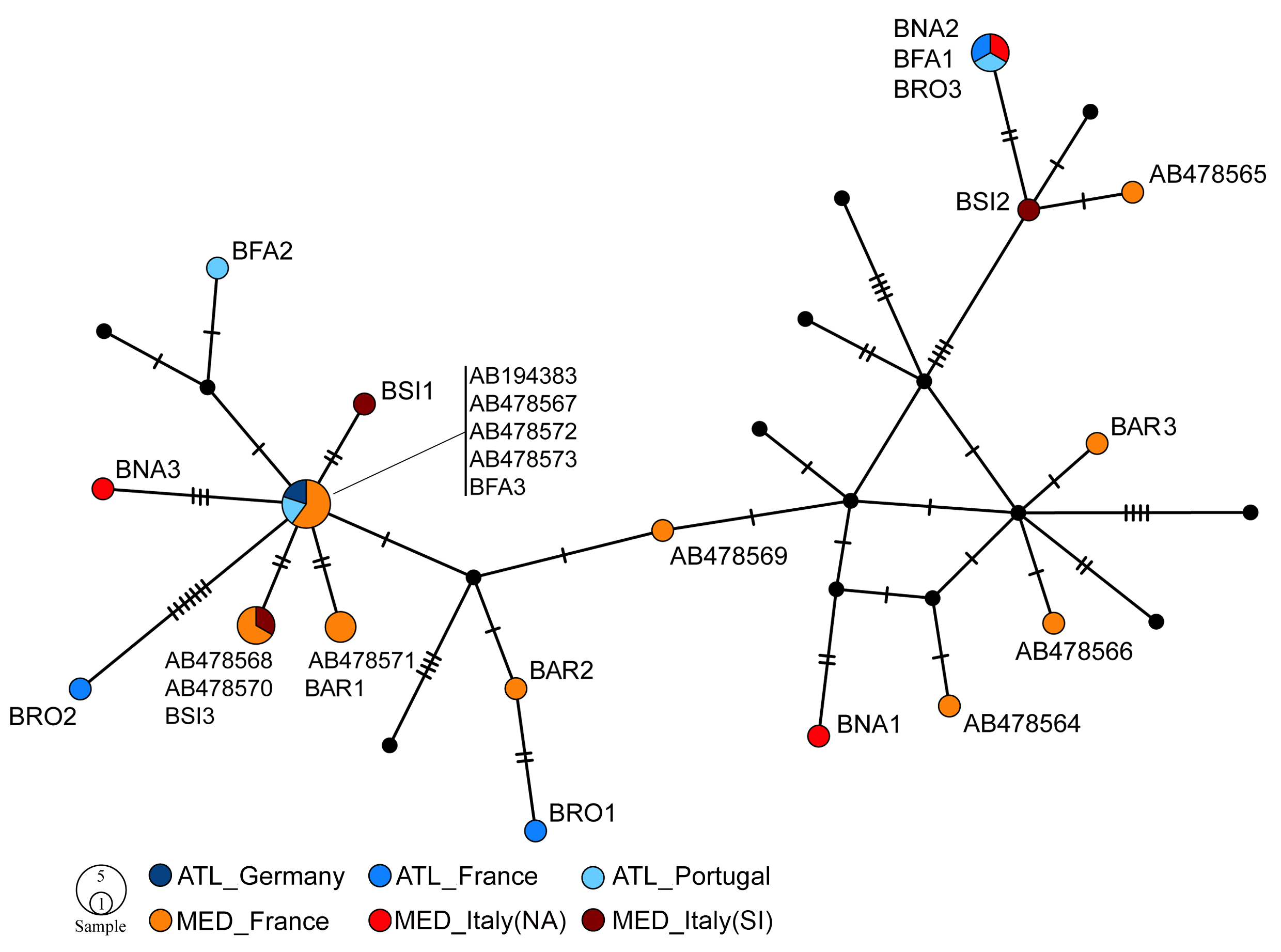

Supplement: S2 Fig — See S1 Table for codes. Abbreviations used: ATL–Atlantic Ocean; MED–Mediterranean Sea; NA–Napoli; SI–Siracusa. Circles representing haplotypes are scaled to their frequencies. Black dots represent missing intermediate haplotypes. Branch length connecting the different haplotypes is proportional to the number of mutations, with small transversal lines along the connecting branches representing mutational steps. (TIF) [file pone.0251358.s004.tif]
